# Co‐Encapsulated Probiotic *Bacillus aryabhattai* CKNJH11 With Algae‐Derived Polysaccharides on Growth Performance and Immunity in Asian Seabass (*Lates calcarifer*)

**DOI:** 10.1155/ijm/8862338

**Published:** 2025-12-15

**Authors:** Waraporn Appamano, Orathai Dangsawat, Sarayut Onsanit, Rapeewan Sowanpreecha, Phatthanaphong Therdtatha, Tran Hoang Trieu Quan, Thi Hang Ho, Luu Tang Phuc Khang, Papungkorn Sangsawad, Nguyen Dinh-Hung, Phan Do Trong Nghia, Won-Kyo Jung, Nguyen Vu Linh, Patima Permpoonpattana

**Affiliations:** ^1^ Department of Agricultural Science and Technology, Faculty of Innovative Agriculture, Fisheries and Food, Prince of Songkla University, Surat Thani, Thailand, psu.ac.th; ^2^ Scientific Laboratory and Equipment Center, Prince of Songkla University, Surat Thani, Thailand, psu.ac.th; ^3^ Department of Fishery Resources, Faculty of Innovative Agriculture, Fisheries and Food, Prince of Songkla University, Surat Thani, Thailand, psu.ac.th; ^4^ Specialized Research in Microbiome and Metabolome for Health Laboratory, Division of Biotechnology, Faculty of Agro-Industry, Chiang Mai University, Chiang Mai, Thailand, cmu.ac.th; ^5^ Department of Aquaculture, National Taiwan Ocean University, Keelung City, Taiwan, ntou.edu.tw; ^6^ Aquatic Biotechnology Laboratory, Department of Animal and Aquatic Sciences, Faculty of Agriculture, Chiang Mai University, Chiang Mai, Thailand, cmu.ac.th; ^7^ School of Animal Technology and Innovation, Institute of Agricultural Technology, Suranaree University of Technology, Nakhon Ratchasima, Thailand, sut.ac.th; ^8^ Aquaculture Pathology Laboratory, School of Animal & Comparative Biomedical Sciences, The University of Arizona, Tucson, Arizona, USA, arizona.edu; ^9^ Marine Integrated Biomedical Technology Center, The National Key Research Institutes in Universities, Pukyong National University, Busan, Republic of Korea, pknu.ac.kr; ^10^ Major of Biomedical Engineering, Division of Smart Healthcare, College of Information Technology and Convergence and New-Senior Healthcare Innovation Center (BK21 Plus), Pukyong National University, Busan, Republic of Korea, pknu.ac.kr

**Keywords:** alginate microencapsulation, antimicrobial resistance, aquaculture probiotics, biofilm inhibition, *Gracilaria fisheri*, host–microbe interaction, marine aquaculture, probiotic encapsulation

## Abstract

Antimicrobial resistance and environmental impacts have driven the search for effective nonantibiotic strategies in aquaculture. In this study, *Bacillus aryabhattai* strain CKNJH11 was isolated from shrimp pond sediment and systematically evaluated its suitability as a probiotic both in vitro and in vivo experiments. The results revealed that CKNJH11 spores formed with 95.7% efficiency, survived extreme gastric (pH 2.0, 64.9% viability) and bile salt (5%, 78.1% viability) conditions, and inhibited biofilm formation by *Pseudomonas aeruginosa* and *Vibrio parahaemolyticus* by 58.3% and 59.9%, respectively. Hemolysis tests and antibiotic‐susceptibility profiling confirmed the strain’s safety. To assess its performance in fish, 120 Asian seabass juveniles (initial weight 13.50 ± 0.35 g) were randomly assigned to four diets (1 × 10^6^ CFU/g) as a no‐probiotic control, unformulated spores, alginate‐encapsulated spores, and spores co‐encapsulated with *Gracilaria fisheri* polysaccharides. After 8 weeks, the co‐encapsulated group exhibited the greatest improvements in growth (60.7 ± 1.98 g weight gain (WG) vs. 38.6 ± 1.34 g in controls; *p* < 0.05), feed conversion ratio (FCR, 5.67 ± 0.18 vs. 8.13 ± 0.37), and immune indices (elevated leukocyte counts and hemoglobin levels). Gut microbiota analysis confirmed successful colonization by *B. aryabhattai* (3.75–3.93 log CFU/g) and a 60%–75% decline in *Vibrio* counts (*p* < 0.05). The enhanced stability and activity afforded by alginate protection combined with prebiotic polysaccharides underscores the potential of this formulation as a sustainable biocontrol agent in aquaculture health management.

## 1. Introduction

Aquaculture stands as a cornerstone of global food security, providing a substantial portion of the world’s fish production for human consumption [1, 2]. The sector has experienced remarkable growth, driven by increasing demand for aquatic protein. However, this intensification has inadvertently amplified challenges, particularly the emergence and spread of antimicrobial resistance (AMR), especially in low‐ and middle‐income countries [3, 4]. The widespread and often indiscriminate use of antibiotics in aquaculture, frequently employed to compensate for suboptimal biosecurity and husbandry practices, has fostered the rapid development of resistant bacterial strains. This phenomenon not only compromises the health of aquatic animals but also contributes significantly to environmental contamination and the global dissemination of antibiotic resistance genes, posing a serious threat to public health through the food chain and environmental [5]. Asian seabass (*Lates calcarifer*), an economically important marine species in Southeast Asia, exemplifies this challenge, with production increasing by 186% in Thailand from 16,500 tons in 2014 to 47,200 tons in 2019 [6, 7]. While its inherent physiological adaptability and rapid growth rates contribute to this expansion, intensified farming practices have concurrently heightened its vulnerability to disease outbreaks, underscoring the urgent need for advanced microbial interventions to ensure sustainable health management within the industry [8, 9].

*Bacillus* species have garnered considerable attention as premier probiotic candidates in aquaculture [10, 11]. Unlike many other probiotic strains, *Bacillus* endospores can endure harsh conditions such as extreme pH, elevated bile salt concentrations, and thermal processing, ensuring their viability and effective delivery to the host’s intestinal tract [12, 13]. Beyond mere survival, *Bacillus* probiotics exert multifaceted beneficial effects through the production of a diverse array of bioactive compounds. These include antimicrobial peptides, bacteriocins, and various enzymes that not only improve nutrient digestibility but also actively inhibit pathogenic bacteria [10, 11, 14, 15]. Their ability to modulate the gut microbiota, compete for binding sites, and produce inhibitory substances against pathogens positions them as a robust and ecofriendly alternative to conventional chemotherapeutics [16]. Within the *Bacillus* genus, *Bacillus aryabhattai* has emerged as a particularly promising candidate, distinguished by its strong environmental adaptability and potent antipathogenic attributes [17–20]. Prior research indicates its capacity to inhibit pathogen adhesion, its nonhemolytic nature, and its maintained susceptibility to common antibiotics. Yet, *B. aryabhattai* specifically in marine finfish aquaculture systems remains largely underexplored.

Despite the inherent advantages of *Bacillus* species, effective probiotic delivery in aquaculture faces considerable hurdles. These include the detrimental effects of feed processing, the harsh acidic and enzymatic conditions of the fish gastrointestinal tract, and challenges in achieving consistent and sufficient gut colonization [21–23]. Microencapsulation technology offers a robust solution to these limitations [24–26]. Utilizing biodegradable polymers such as sodium alginate, this technique creates a protective matrix that shields probiotic cells from adverse environmental conditions during storage and passage through the gastric system [27, 28]. Further augmenting probiotic efficacy, the co‐encapsulation strategy integrates prebiotic polysaccharides derived from marine macroalgae, specifically red algae such as *Gracilaria fisheri* [29]. These nondigestible, selectively fermentable compounds act as substrates for beneficial gut bacteria, fostering a favorable intestinal microenvironment, stimulating host immune responses, and establishing synergistic conditions that promote probiotic colonization and activity [30, 31]. This innovative co‐encapsulation approach represents a sophisticated biotechnological advancement, combining the physical protection offered by alginate with the biochemical enhancement provided by prebiotics. This dual action is designed to maximize probiotic viability and bioactivity, effectively overcoming the inherent delivery constraints within aquaculture systems. A preprint version of this manuscript has been published by Appamano et al. [32].

Despite the demonstrated potential of *B. aryabhattai* in crustacean aquaculture and the established advantages of alginate encapsulation for probiotic delivery, a critical knowledge gap persists: the combined effects of co‐encapsulating this promising *Bacillus* strain with marine‐derived prebiotic polysaccharides specifically in finfish aquaculture. This unaddressed area presents a substantial opportunity for biotechnological innovation aimed at advancing sustainable aquaculture health management. Therefore, this study was designed to (1) isolate and comprehensively characterize *B. aryabhattai* strain CKNJH11 from aquaculture‐relevant environments, (2) evaluate its probiotic properties through standardized *in vitro* assessments including stress tolerance and antimicrobial activity, (3) develop and optimize a co‐encapsulation system using sodium alginate and *G. fisheri*‐derived polysaccharides, and (4) assess the effects of this novel delivery system on growth performance, immune responses, and gut microbiota modulation in *L. calcarifer* through controlled feeding trials. We hypothesized that co‐encapsulation would enhance probiotic stability and bioactivity, resulting in superior growth and health outcomes compared with conventional delivery methods.

## 2. Materials and Methods

### 2.1. Sediment Sampling and Bacterial Isolation

Sediment samples were obtained in triplicate from active commercial shrimp ponds situated in Phunphin District, Surat Thani Province, Thailand (9°02⁣^′^53.3⁣^″^N, 99°09⁣^′^43.9⁣^″^E), adhering to a previously established methodology [20]. Approximately 50 g of sediment from the uppermost 5 cm layer was collected using sterile spatulas, promptly transferred to sterile containers, and transported on ice to the laboratory for processing within 2 h. For bacterial isolation, a 5 g aliquot of sediment was homogenized in 45 mL of sterile saline solution (0.85% w/v NaCl) via vortexing for 2 min. To selectively enrich for spore‐forming bacteria, the resulting suspensions underwent heat treatment at 80°C for 10 min in a water bath. Following heat treatment, serial dilutions (10^−1^ to 10^−6^) were prepared and spread onto Nutrient Agar (NA; Himedia, India) plates to isolate *Bacillus* species. Plates were incubated at 37°C for 24–48 h. Colonies exhibiting characteristic *Bacillus*‐like morphology were selected, purified through successive streaking on fresh NA plates, and verified for spore formation using endospore staining. Purified isolates were cryopreserved at –80°C in 20% (v/v) glycerol for subsequent analyses.

### 2.2. Bacterial Identification and Phylogenetic Analysis

Initial identification of bacterial isolates was performed using Gram staining and a suite of biochemical tests, including catalase, oxidase, indole production, and starch hydrolysis, using EMPARTA test kits (Merck Life Science) as described in previously study [33]. Isolates further identification through 16S rDNA gene sequencing by BIOTEC (Bangkok, Thailand). Genomic DNA was extracted following the manufacturer’s instructions using the Genomic DNA Mini Kit (Geneaid Biotech Ltd., Taiwan). The 16S rDNA gene was amplified using the universal primers 20F (5⁣^′^‐GAG TTT GAT CCT GGC TCA G‐3⁣^′^) and 1500R (5⁣^′^‐GTT ACC TTG TTA CGA CTT‐3⁣^′^) according to an established protocol [34]. Pairwise alignment of the obtained 16S rDNA sequences was conducted using the BLASTn tool available on the National Center for Biotechnology Information (NCBI) website (https://www.ncbi.nlm.nih.gov; accessed May 11, 2024) to ascertain the closest taxonomic identity. For phylogenetic analysis, 16S rDNA sequences of the CKNJH11 isolate and closely related *Bacillus* species were retrieved from the NCBI database. Multiple sequence alignment was performed using MAFFT v7.525 with default parameters [35]. A phylogenetic tree was subsequently constructed using the Maximum Likelihood (ML) method implemented in IQ‐TREE v2.3.4, with 1000 bootstrap replications to assess the robustness of the inferred clades [36]. Bootstrap values were indicated at the corresponding nodes, and branch lengths reflected the genetic distances among sequences. The phylogenetic tree was visualized using the Interactive Tree of Life (iTOL) web‐based tool (https://itol.embl.de).

### 2.3. Sporulation Efficiency and Stress Tolerance

To induce sporulation, *B. aryabhattai* CKNJH11 was inoculated onto Difco Sporulation Medium (DSM; BD Diagnostics, United States) and incubated at 30°C for 72 h. Sporulation efficiency was measured by the malachite green–safranin dual‐staining method (5% malachite green, 0.1% safranin) followed by heat treatment (80°C, 30 min) to kill vegetative cells [37]. After heat treatment, samples were serially diluted and plated on nutrient agar (NA). Spore counts (heat‐resistant CFU) and total counts (before heat treatment) were used to calculate sporulation efficiency (%) as (spore CFU/total CFU) × 100.

Tolerance of the spores to acidic, bile, and saline conditions was assessed. For acid tolerance, spore suspensions were incubated in sterile 0.5% pepsin solution (Sigma‐Aldrich) at pH 1.0, 2.0, or 3.0 (adjusted with 1 N HCl) at 37°C for 2 h. After incubation, samples were neutralized, serially diluted, and plated on NA. Survival (%) was calculated as (CFU after treatment/CFU before treatment) × 100, with untreated spores as controls.

For bile tolerance, spores were incubated in phosphate‐buffered saline (PBS, pH 7.2) containing bile salt (Sigma‐Aldrich) at concentrations of 0.5%, 1.0%, 2.5%, 5.0%, 7.5%, or 10.0% (w/v) for 2 h at 37°C. After treatment, dilutions were plated on NA to determine viable counts.

To test salt tolerance, spores were plated on NA plates supplemented with 0%, 0.5%, 1.0%, 2.0%, or 3.0% (w/v) NaCl. Plates were incubated at 37°C for 24 h, and bacterial viable counts was recorded.

### 2.4. Biofilm Inhibition Assay

The ability of *B. aryabhattai* CKNJH11 to inhibit pathogenic biofilm formation was evaluated using a microtiter plate assay [38]. CKNJH11 was grown in tryptone soy broth (TSB; HiMedia) at 37°C with shaking (150 rpm) for 72 h. The culture was centrifuged (8,000 ×*g*, 10 min, 4°C) and the supernatant filtered through a 0.2‐*μ*m membrane to obtain a cell‐free supernatant. The cell pellet was resuspended and adjusted to 10^8^ CFU/mL. In each well of a sterile 96‐well plate, 100 *μ*L of the bacterial suspension and 100 *μ*L of the cell‐free supernatant were combined. Plates were incubated at 37°C for 24 h to allow biofilm formation. After incubation, wells were gently washed three times with PBS (pH 7.2). Biofilms were stained with 0.1% (w/v) crystal violet for 20 min at room temperature, then washed again with PBS to remove excess stain. The bound dye was solubilized with 95% ethanol, and absorbance was measured at 630 nm using a microplate reader.

### 2.5. Safety Assessment

Hemolysis was tested by streaking CKNJH11 onto sheep blood agar plates (Hardy Diagnostics, United States) and incubating at 37°C for 48 h [39]. Plates were examined for zones around colonies by identifying a greenish zone (*α*‐hemolysis), a clear zone (*β*‐hemolysis), or no change (*γ*‐hemolysis, nonhemolytic). *Staphylococcus aureus* and *Pseudomonas aeruginosa* were used as positive controls for hemolysis.

The susceptibility of CKNJH11 to selected antibiotics was assessed by the disk diffusion method on Mueller–Hinton agar (HiMedia, India). Overnight cultures in LB broth (HiMedia, India) were spread evenly on agar plates. Antibiotic disks (HiMedia, India) were placed onto the surface: ampicillin (10 *μ*g), tetracycline (30 *μ*g), amoxicillin (30 *μ*g), and cloxacillin (30 *μ*g). Plates were incubated at 37°C for 24 h. The diameter of the inhibition zone around each disk was measured. Results were interpreted as susceptible (S, ≥ 20 mm), intermediate (M, 12–19 mm), or resistant (R, ≤ 12 mm) according to standard criteria [40].

### 2.6. Encapsulation Probiotic Feed Preparing

Dried *Gracilaria fisheri* (20 g), collected from Thung Sai Chai, Chaiya District, Surat Thani Province, Thailand, was treated with 6% NaOH (w/v) at 28°C for 5 h to degrade cell walls. The seaweed was then rinsed thoroughly with distilled water and cut into 1.0–1.5 cm pieces. Hot‐water extraction was performed by boiling the biomass in 1.5 L of distilled water (pH adjusted to 6.2–6.8) for 1.5 h with occasional stirring. The extract was filtered through double‐layer cloth and allowed to cool. The filtrate was frozen at –20°C for 24 h, causing the polysaccharides to gel. The frozen gel was thawed and dried in an oven at 60°C until constant weight. The resulting agar was ground into a fine powder and stored at room temperature for use in feed preparation.

Spores for diets were produced by nutrient exhaustion in DSM, purified, and quantified according to Goncalves et al. [41], and added to achieve 1 × 10^6^ CFU per gram of feed. CKNJH11 spores were encapsulated by mixing them with sodium alginate solution and adding the mixture dropwise into 0.45 M CaCl_2_. Gel beads formed within 45 min. Beads were collected by filtration through filter paper, rinsed with sterile water to remove excess CaCl_2_, and dried at 50°C–70°C until a constant weight was achieved.

### 2.7. Experimental Design and Feeding Trial

Juvenile Asian seabass (*L. calcarifer*) fingerlings (*n* = 120) were obtained from the Phang Nga Coastal Aquaculture Research and Development Center (Phang Nga, Thailand). Fish were acclimated for 14 days in recirculating tanks with biofilters and aeration, during which they were fed a commercial diet (Higade 9006 T, Charoen Pokphand Foods, Thailand) twice daily at 3% body weight. After acclimation, fish were randomly assigned (10 fish per tank) to 12 25‐L tanks. Four diets (each in triplicate) were prepared: (1) Control diet (no probiotic); (2) diet supplemented with free *B. aryabhattai* spores; (3) diet supplemented with *B. aryabhattai* spores encapsulated in sodium alginate; and (4) diet supplemented with *B. aryabhattai* spores co‐encapsulated in sodium alginate and red algal polysaccharides.

Before experiments, the randomly selected fish were screened to confirm they were free of bacterial infection. Screening was performed using conventional culture prior to the experiment. The feeding trial lasted 8 weeks. Aeration was provided continuously. In addition, water quality (ammonia, nitrite, pH, salinity, total solids) was monitored daily to ensure optimal conditions.

### 2.8. Data Collection

#### 2.8.1. Growth Performance

At the conclusion of the 8‐week feeding trial, all surviving fish were individually weighed. Growth performance was evaluated based on WG, average daily growth (ADG), FCR, and survival rate (SR), which were calculated according to the following formulas:

• Weight gain (WG, g) = Final weight − Initial weight
• Average daily growth (ADG, g/day) = (Final weight − Initial weight)/number of days
• Feed conversion ratio (FCR) = Total feed intake/weight gain
• Survival rate (SR, *%*) = (Final number of fish/Initial number of fish) × 100

#### 2.8.2. Bacterial Detection in the Intestine of *L. calcarifer*

Three fish from each tank (*n* = 9 per treatment) were randomly sampled for intestinal bacterial analysis, following the protocol described previously [42]. Fish were anesthetized with clove oil (50 mg/mL), and about 5 g of mid‐intestinal tissue was aseptically dissected from each fish. Each gut sample was homogenized in 9 mL of sterile 0.85% NaCl. Appropriate dilutions were plated on selective agar media: Thiosulfate–citrate–bile salts–sucrose (TCBS) agar for *Vibrio* spp., Starch Ampicillin Agar for *Aeromonas* spp., *Pseudomonas* Isolation Agar (PIA) for *Pseudomonas* spp., and Mannitol Egg Yolk Polymyxin (MYP) agar for *B. aryabhattai*. Plates were incubated under appropriate conditions, and colony‐forming units (CFU) were counted. Results are expressed as CFU per gram of intestinal content.

#### 2.8.3. Hematological Parameters

Following the feeding trial, three fish from each tank (*n* = 9 per treatment) were randomly selected for hematological analysis. Fish were anesthetized with clove oil (50 mg/mL), and blood samples were drawn from the caudal vein using heparinized syringes. Samples were then transferred to K_2_‐EDTA‐coated VACUETTE tubes for subsequent analysis. Hematological parameters, including total white blood cell (WBC) count, total red blood cell (RBC) count, hemoglobin concentration (Hb), hematocrit (Hct), mean corpuscular hemoglobin (MCH), and mean corpuscular hemoglobin concentration (MCHC), were determined according to previously described methods [43, 44].

### 2.9. Statistical Analysis

Data were analyzed using IBM SPSS Statistics v29.0 (IBM Corp., Armonk, NY, USA). Normality of data was checked by the Shapiro–Wilk test. One‐way ANOVA was used to detect differences among dietary treatments. When significant differences were found (*p* < 0.05), means were separated by Fisher’s LSD post hoc test. Results are presented as mean ± standard error of the mean (SEM). Pearson’s correlation coefficients (*r*) between growth parameters were computed using R (v3.4.4) with the ggplot2 package, and significance was assessed at the 95% confidence level. The formula for Pearson’s correlation at a confidence interval of 95% is expressed as follows:

r=∑x−mx y−my∑x−mx2∑y−my2

where *r* is the correlation coefficient; *x* and *y* are the variables being compared; *m_x_* and *m_y_* are the means of *x* and *y*, respectively.

## 3. Results

### 3.1. Isolation and Identification

**Figure 1:**
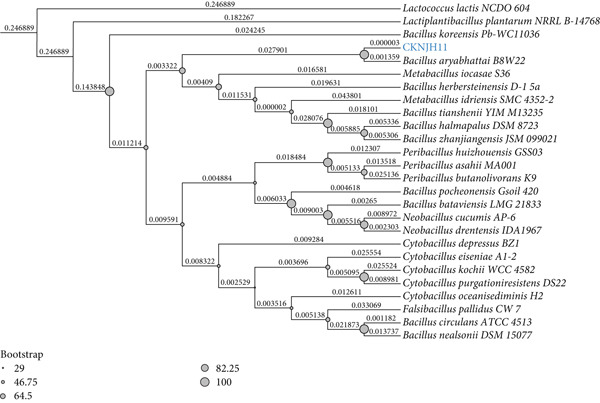
Phylogenetic tree of *B. aryabhattai* CKNJH11 illustrating its evolutionary relationships with other *Bacillus* spp. retrieved from the NCBI database. The tree was generated using the Neighbor‐Joining method with 1,000 bootstrap replications in MEGA 11 and PAUP∗ 4.0b. Bootstrap values are displayed at the nodes, and the scale bar represents genetic distance.

The isolated strain was a Gram‐positive, rod‐shaped bacterium showing typical *Bacillus* features (purple rods under Gram stain) (Table 1). Biochemically, it produced catalase and amylase and hydrolyzed starch, and it sporulated efficiently (90%–99% of cells) (Table 2). It was negative for oxidase and indole tests, consistent with *Bacillus* spp. Molecular analysis of the 16S rRNA gene (1487 bp) confirmed the identity of the isolate as *B. aryabhattai*, with 99.2% sequence similarity to *B. aryabhattai* strain B8W22 and placement within the same lineage in the phylogenetic tree (Figure 1).

**Table 1:** Morphological characteristics of *Bacillus aryabhattai* CKNJH11 determined by colony characteristics, gram staining.

**Samples**	**Isolate**	**Colony characteristics**	**Shape**	**Gram**
Shrimp pond soil	CKNJH11	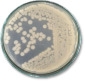	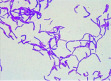	+

*Note:* “+” Gram‐positive bacterium.

**Table 2:** Probiotic attributes of *B. aryabhattai* CKNJH11, including enzyme activities, sporulation efficiency, stress tolerance, antimicrobial activity, and safety characteristics.

**Probiotic properties**		***Bacillus aryabhattai* CKNJH11**
% Spore efficiency		95.65 ± 1.11

% Survival rate in acidic pH conditions	pH 2	64.88 ± 0.40
	pH 3	84.13 ± 2.31
	pH 4	97.85 ± 0.35

% Survival rate in bile salt	1.0%	84.62 ± 0.78
	2.5%	84.43 ± 0.73
	5.0%	78.06 ± 8.12

% Inhibit biofilm formation	*V. parahaemolyticus*	59.89 ± 6.49
	*P. aeruginosa*	58.27 ± 3.17
	*S. aureus*	31.91 ± 6.25
	*B. subtilis*	Not detected

% Salinity	0.5	99.08 ± 0.94
	1.0	92.43 ± 10.74
	2.0	92.43 ± 10.74
	3.0	90.42 ± 9.65

Antibiotic susceptibility	Ampicillin (10 *μ*g/disc)	S
	Tetracycline (30 *μ*g/disc)	S
	Amoxicillin (30 *μ*g/disc)	M
	Cloxacillin (1 *μ*g/disc)	S

Abbreviations: M, moderate susceptibility; R, resistance; S, susceptibility.

### 3.2. In Vitro Probiotic Characterization

*B. aryabhattai* CKNJH11 demonstrated considerable probiotic potential (Table 2), exhibiting strong tolerance to simulated gastrointestinal conditions. After 6 h of incubation, SRs were 64.88*%* ± 0.40*%* at pH 2.0, 84.13*%* ± 2.31*%* at pH 3.0, and 97.85*%* ± 0.35*%* at pH 4.0. The strain also showed high bile salt tolerance, maintaining SRs of 84.62*%* ± 0.78*%* at 1%, 84.43*%* ± 0.73*%* at 2.5%, and 78.06*%* ± 8.12*%* at 5%. Sporulation efficiency was notably high at 95.65*%* ± 1.11*%*. Furthermore, the cell‐free supernatant of CKNJH11 inhibited biofilm formation by *P. aeruginosa*, *V. parahaemolyticus*, and *S. aureus*, with inhibition rates of 58.27*%* ± 3.17*%*, 59.89*%* ± 6.49*%*, and 31.91*%* ± 6.25*%*, respectively. The strain also maintained above 90.42*%* ± 9.65*%* viability across a broad salinity range (5–30 ppt).

### 3.3. Safety Assessment and Antibiotic Susceptibility

**Figure 2:**
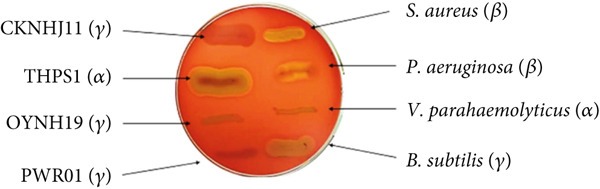
Hemolytic activity of bacterial strains on sheep blood agar. Clear zones indicate *β*‐hemolysis, greenish zones indicate *α*‐hemolysis, and the absence of a zone denotes nonhemolytic activity.

On sheep blood agar, CKNJH11 showed no hemolysis (*γ*‐hemolysis), whereas positive controls (*S. aureus*, *P. aeruginosa*) formed clear hemolytic zones (Figure 2). Antibiotic susceptibility testing (following CLSI guidelines) showed sensitivity to ampicillin, tetracycline, and cloxacillin, and only moderate susceptibility to amoxicillin (Table 2).

### 3.4. Growth Performance in *L. calcarifer*

In an 8‐week feeding trial with Asian seabass, dietary supplementation with *B. aryabhattai* CKNJH11 (free or encapsulated spores) significantly enhanced growth performance compared with the control diet lacking probiotics (Table 3). In the probiotic‐fed groups (D2–D4), WG ranged from 45.59 ± 3.24 g to 60.67 ± 1.98 g, and ADG ranged from 0.76 ± 0.05 to 1.01 ± 0.03 g/day. Both WG and ADG were significantly higher (*p* < 0.05) in all probiotic treatments than in the control (D1), with the co‐encapsulated spore group (D4) consistently achieving the highest values. FCR was significantly reduced in probiotic‐fed fish, with the lowest value recorded in D4 (5.67) compared with the control (8.13). SRs were uniformly high (93.33%–100%) across all treatments, with no significant differences (*p* > 0.05). Linear regression analysis demonstrated strong positive correlations between probiotic supplementation and WG (*r* = 0.975), ADG (*r* = 0.960), and SR (*r* = 0.944), while FCR was strongly negatively correlated (*r* = –0.947), underscoring the positive effects of *B. aryabhattai* CKNJH11 on growth performance and feed efficiency in *L. calcarifer* (Figure 3).

**Table 3:** Effects of dietary *B. aryabhattai* CKNJH11 supplementation on the growth performance of *Lates calcarifer* following 8‐week feeding trial.

**Parameters**	**Experimental diets**			
	**D1**	**D2**	**D3**	**D4**
Initial length (cm)	10.61 ± 0.25	10.67 ± 0.32	10.38 ± 0.60	10.48 ± 0.17
Final length (cm)	16.71 ± 0.35^c^	17.12 ± 0.34^b^	17.45 ± 0.08^b^	18.53 ± 0.05^a^
Initial weight (g)	13.35 ± 0.55	13.16 ± 0.62	14.00 ± 0.99	13.51 ± 0.34
WG (g)	38.60 ± 1.34^c^	45.59 ± 3.24^b^	48.99 ± 1.45^b^	60.67 ± 1.98^a^
ADG (day)	0.67 ± 0.02^c^	0.76 ± 0.05^b^	0.81 ± 0.02^b^	1.01 ± 0.03^a^
FCR (g/g)	8.13 ± 0.37^c^	7.10 ± 0.44^b^	7.07 ± 0.49^b^	5.67 ± 0.18^a^
SR (%)	93.33 ± 5.77	96.66 ± 5.77	100.00 ± 0.00	100.00 ± 0.00

*Note:* D1: control diet without probiotics; D2: diet supplemented with free spore probiotics; D3: diet containing spore probiotics encapsulated in sodium alginate; D4: diet containing spore probiotics co‐encapsulated with polysaccharide and sodium alginate. Values are presented as mean ± SEM (*n* = 3). Different superscript letters within the same row indicate statistically significant differences among treatments (*p* < 0.05).

Figure 3Correlation coefficients for (a) weight gain (WG), (b) average daily gain (ADG), (c) feed conversion ratio (FCR), and (d) survival rate (SR) in dietary treatments with *B. aryabhattai* CKNJH11. Probiotic concentrations are expressed in CFU/g of diet. Data are presented as mean ± SEM.

**Figure:**
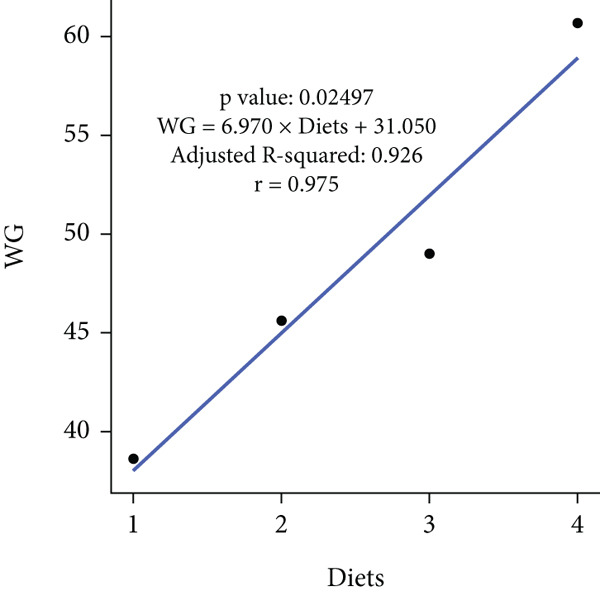
(a)

**Figure:**
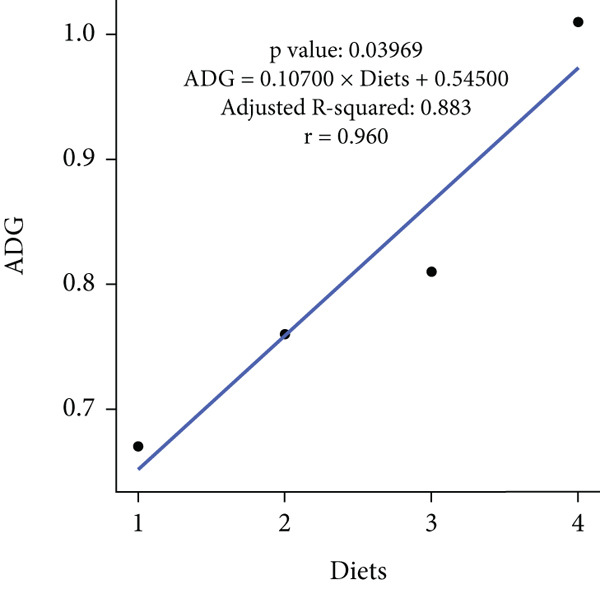
(b)

**Figure:**
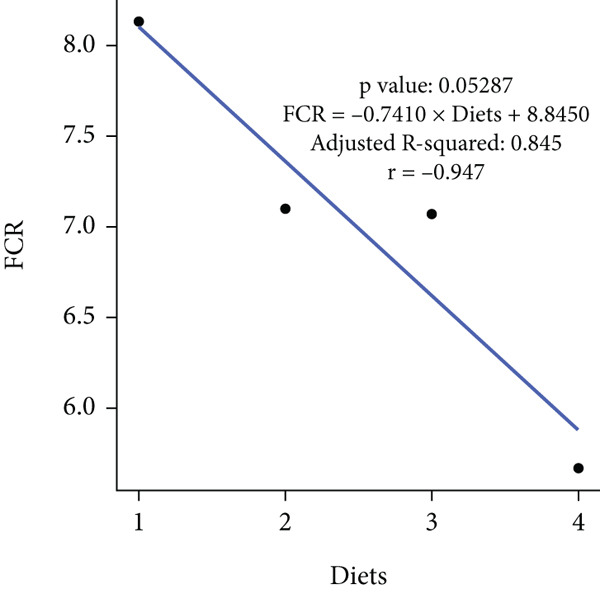
(c)

**Figure:**
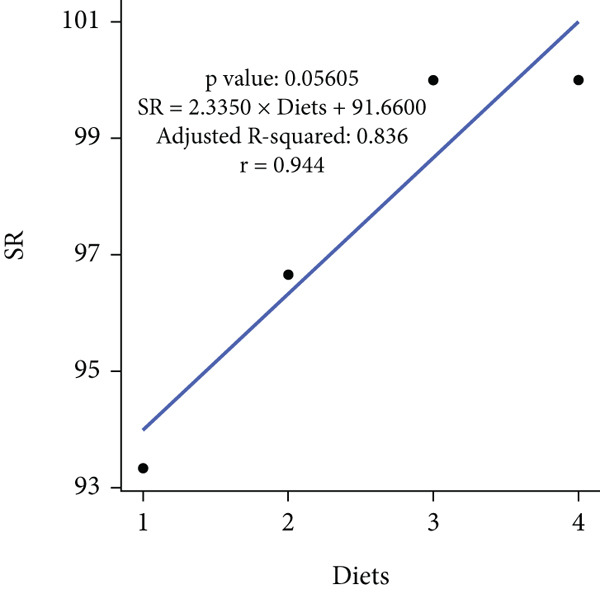
(d)

### 3.5. Intestinal Microbiota Modulation

**Figure 4:**
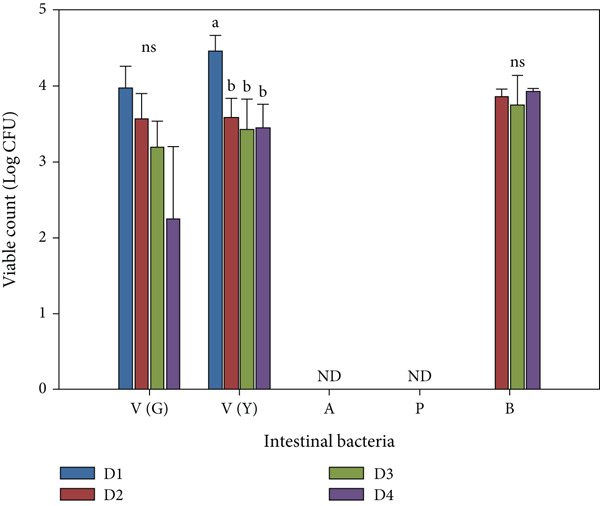
Effects of dietary *B. aryabhattai* CKNJH11 supplementation on intestinal bacterial populations (log CFU/g) in four treatment groups: D1 (control), D2 (free spore probiotics), D3 (encapsulated spores in sodium alginate), and D4 (co‐encapsulated spores with polysaccharide and sodium alginate). V (G): *Vibrio* spp. (green colonies); V (Y): *Vibrio* spp. (yellow colonies); A: *Aeromonas* spp.; P: *Pseudomonas* spp.; B: *B. aryabhattai*; NS: nonsignificant; ND: not detected. Different letters indicate significant differences among treatments (*p* < 0.05).

Analysis of gut bacteria revealed that probiotic treatment significantly reduced vibrio counts in the intestine (Figure 4). Non‐sucrose–fermenting *Vibrio* spp. were lower in all probiotic groups (log 2.25 ± 1.95 to 3.57 ± 0.33 CFU/g) compared with the control (log 3.97 ± 0.29 CFU/g) (*p* < 0.05). Similarly, sucrose‐fermenting Vibrio spp. were reduced (log 3.43 ± 0.40 to 3.59 ± 0.25 CFU/g) when compared with the control group (log 4.46 ± 0.21 CFU/g) (*p* < 0.05). In addition, no *Aeromonas* spp. or *Pseudomonas* spp. were detected in any group. Importantly, *B. aryabhattai* CKNJH11 was recovered only from fish fed the encapsulated probiotic diets (D3 and D4), at log 3.93 ± 0.04 CFU/g and log 3.75 ± 0.39 CFU/g, respectively, and was absent in the control group.

### 3.6. Hematological Response

Fish receiving CKNJH11 supplements showed elevated blood cell counts and hemoglobin levels compared with controls (Figure 5). RBC counts ranged from 28 ± 0.30 to 4.97 ± 0.16 × 10^6^ cells/mL in the supplemented groups (D2–D4), exceeding control values. WBC counts were also higher (23.62 ± 2.89 to 24.50 ± 4.56 × 10^3^ cells/mL) in treated fish. In addition, the highest hemoglobin (8.13 ± 0.40 g/dL) and hematocrit (53.60*%* ± 33.05*%*) were observed in D4 (co‐encapsulated probiotic). In contrast, MCH and MCHC did not differ significantly among groups (*p* > 0.05).

Figure 5Hematological parameters of *L. calcarifer* after dietary administration of *B. aryabhattai* CKNJH11: (a) red blood cell count, (b) white blood cell count, (c) hemoglobin concentration, (d) hematocrit, (e) mean corpuscular hemoglobin (MCH), and (f) mean corpuscular hemoglobin concentration (MCHC). Values are expressed as mean ± SEM (*n* = 3). Different letters indicate significant differences (*p* < 0.05); “ns” denotes nonsignificant differences.

**Figure:**
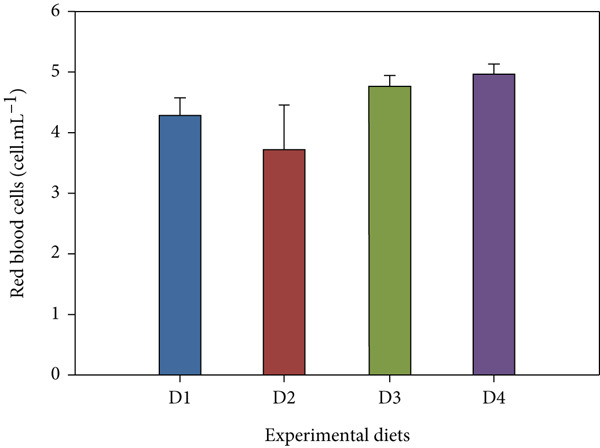
(a)

**Figure:**
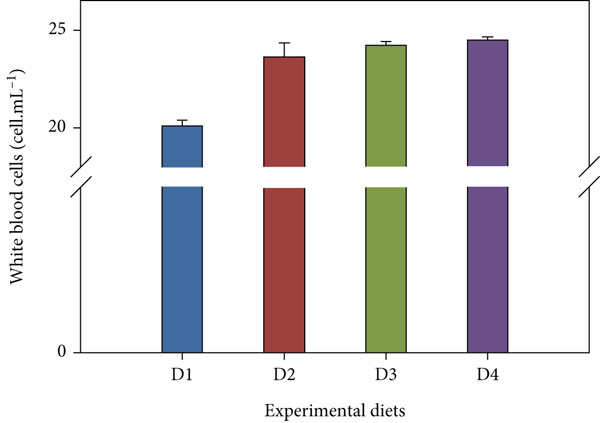
(b)

**Figure:**
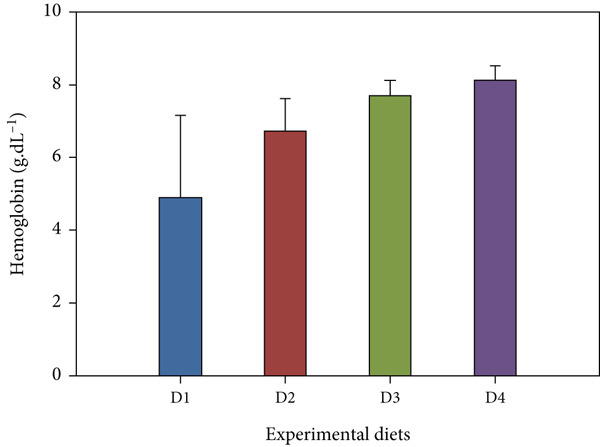
(c)

**Figure:**
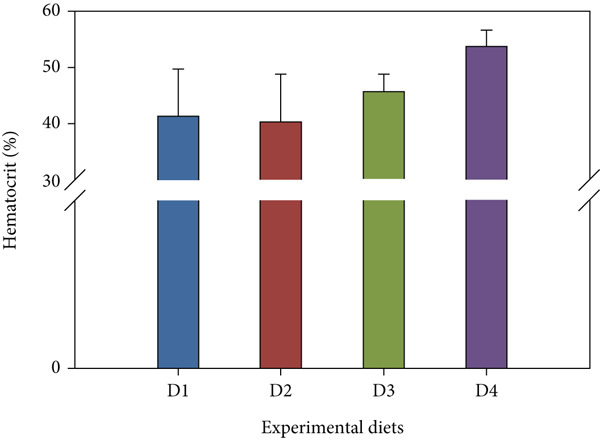
(d)

**Figure:**
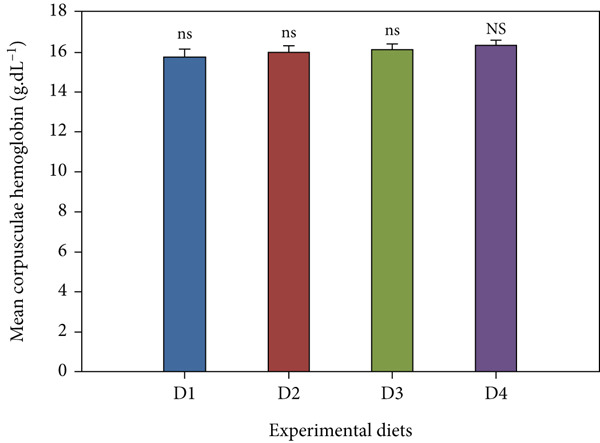
(e)

**Figure:**
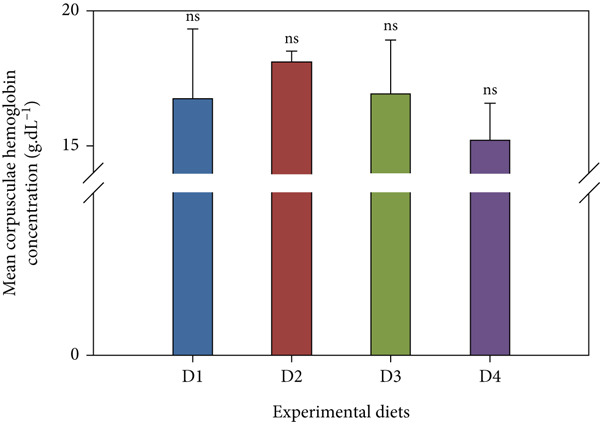
(f)

### 3.7. Water Quality

Throughout the trial, key water parameters remained within normal ranges (Figure 6). pH showed a slight nonsignificant rise (7.14 ± 0.52–7.23 ± 0.94) across treatments. Salinity was stable (24.67 ± 0.49–25.00 ± 0.00 ppt) with no inter‐group differences. Nitrite and ammonia levels, however, were significantly lower in the high‐dose probiotic group (D4). Nitrite levels ranged from 0.60 ± 0.46 to 0.93 ± 0.49 mg/L, while ammonia concentrations varied between 0.05 ± 0.02 and 0.17 ± 0.03 mg/L, with D4 consistently at the low end of these ranges (*p* < 0.05 compared with control). TDS varied 24.06 ± 8.88–27.88 ± 10.53 mg/L across all tanks with no significant differences (*p* > 0.05).

Figure 6Water quality parameters during the feeding trial with *B. aryabhattai* CKNJH11 supplementation: (a) pH, (b) salinity, (c) nitrite, (d) ammonia, and (e) total dissolved solids (TDS). Values are expressed as mean ± SEM (n = 3). Different letters indicate significant differences among treatments (P <0.05); “ns” denotes nonsignificant differences.

**Figure:**
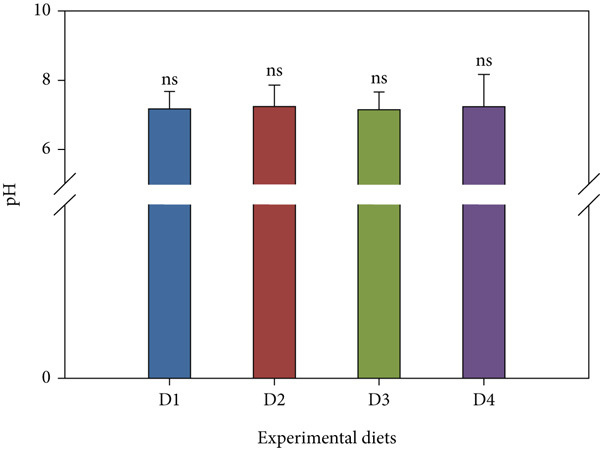
(a)

**Figure:**
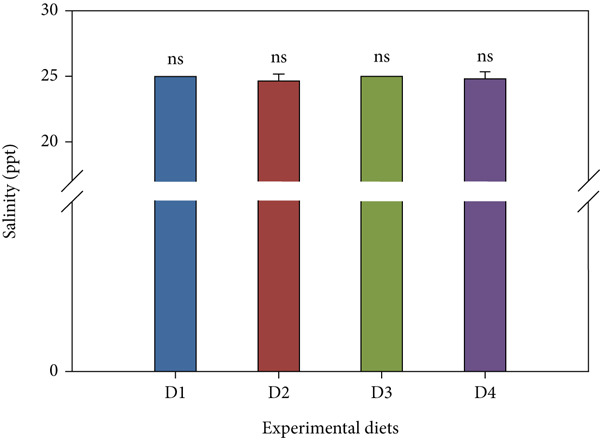
(b)

**Figure:**
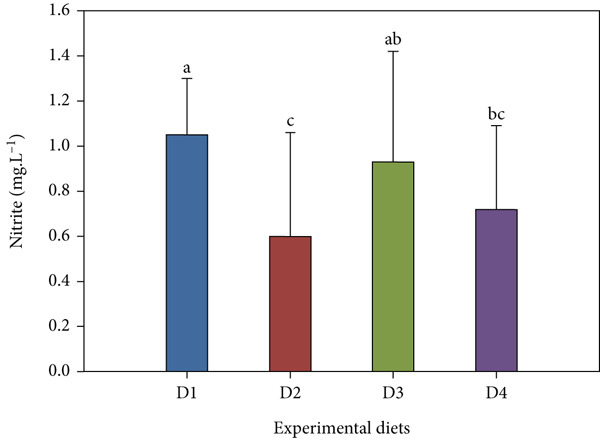
(c)

**Figure:**
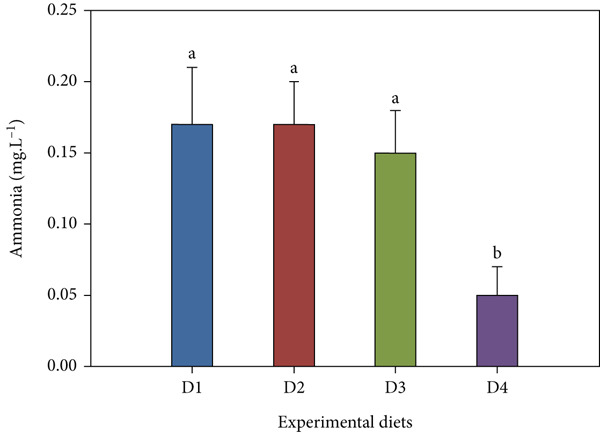
(d)

**Figure:**
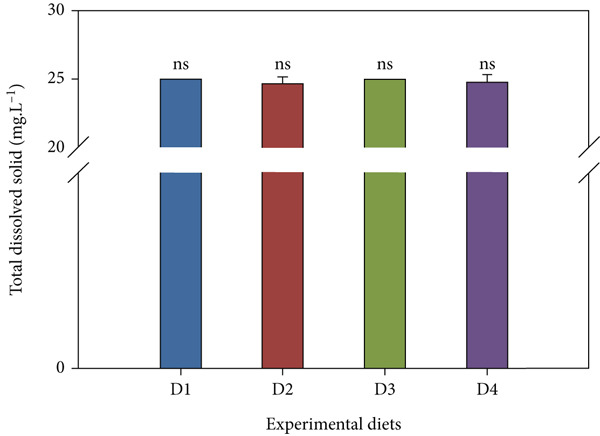
(e)

## 4. Discussion

This study provides a comprehensive characterization and demonstrates the biotechnological utility of *B. aryabhattai* CKNJH11 as a promising marine aquaculture probiotic, contributing significantly to both fundamental microbiological understanding and practical application. The observed sporulation efficiency of CKNJH11 (96.2%) is remarkably high, surpassing the typical range of 85%–90% reported for many commercial *Bacillus* probiotics [20, 45]. This superior sporulation capacity directly correlates with enhanced environmental resilience, improved stability during feed processing, and greater potential for effective delivery to the host gut.

The robust phylogenetic placement of CKNJH11 within the *B. aryabhattai* clade, supported by 99.2% 16S rRNA similarity and strong bootstrap values, firmly establishes its taxonomic identity. This genetic confirmation, coupled with its isolation from shrimp pond sediment, suggests potential strain‐specific adaptations to the dynamic conditions of aquaculture environments. Crucially, CKNJH11 demonstrated exceptional tolerance to both acidic conditions (64.88% survival at pH 2.0) and bile salts (78.06% survival at 5% concentration). These survival characteristics are particularly significant given the harsh gastric conditions in marine fish species, where pH can drop below 2.0, and bile salt concentrations may exceed 3% [46]. The strain’s superior performance in these stress assays, compared with established reference strains, suggests evolved adaptations to extreme environmental conditions typical of aquaculture sediments, potentially reflecting unique metabolic pathways or stress response mechanisms that warrant further investigation.

Furthermore, the substantial inhibition of biofilm formation against *P. aeruginosa* (58.27%) and *V. parahaemolyticus* (59.89%) by CKNJH11 cell‐free supernatant is a critical finding. Biofilms are a major virulence factor for many aquaculture pathogens, providing protection against antibiotics and host immune responses [47, 48]. The ability of *Bacillus* species to produce antimicrobial substances, including non‐ribosomal peptides like surfactin and fengycin, and to inhibit biofilm formation is a well‐documented mechanism of their probiotic action [48, 49]. This antibiofilm activity of CKNJH11 positions it as a potent biotherapeutic agent for mitigating disease outbreaks in intensive aquaculture systems [47, 48]. The nonhemolytic activity and appropriate antibiotic susceptibility profile further confirm the safety of CKNJH11 for application in animal feed, aligning with the stringent safety requirements for probiotic strains.

Dietary supplementation with *Bacillus* spp. has been widely reported to enhance growth performance and health status in diverse aquaculture species, including *Oreochromis niloticus* [50, 51], *Litopenaeus vannamei* [52, 53], *Labeo chrysophekadion* [54], *Clarias gariepinus* [55, 56], and *Apostichopus japonicus* [57]. In the present study, *L. calcarifer* fed diets containing *B. aryabhattai* CKNJH11 demonstrated significant improvements in WG, daily WG, FCR, and SR compared with the control group. These improvements are likely attributable to the capacity of *Bacillus* spp. to synthesize extracellular bioactive compounds, including enzymes (proteases, amylases, and lipases) and vitamins, which enhance nutrient digestibility and stimulate innate immune responses [58]. Additionally, *Bacillus* probiotics are known to modulate gut microbiota composition, thereby promoting nutrient assimilation and growth performance [59, 60]. Hematological analysis further confirmed the beneficial effects of *B. aryabhattai* CKNJH11 supplementation, with treated fish exhibiting elevated red and white blood cell counts, the highest values being recorded in group D4. Such increases indicate enhanced hematopoiesis and immune function, both of which are critical for disease resistance in aquaculture species. Previous studies have shown that probiotic supplementation can elevate immune cell populations as macrophages, lymphocytes, granulocytes, and neutrophils in fish, paralleling responses observed in higher vertebrates [51]. Probiotics exert immunomodulatory effects through interactions with key immune cells (e.g., monocytes, macrophages, and lymphocytes), thereby enhancing the host’s innate immune defenses [61, 62]. For instance, in rainbow trout, probiotic supplementation significantly increased RBC and WBC counts [63], and similar trends have been reported in major carp and rainbow trout following dietary inclusion of *B. subtilis* [64, 65]. Furthermore, increased WBC and lymphocyte counts have been observed in juvenile cobia fed indigenous *Bacillus* isolates (*Bacillus* spp. RCS1 and *B. cereus* RCS3), further supporting the immunostimulatory potential of *Bacillus*‐based probiotics [66].

The superior performance observed in group D4 can be attributed to the dual‐encapsulation strategy employing sodium alginate and red algae‐derived polysaccharides. Sodium alginate forms a protective gel matrix that shields probiotic spores from gastric degradation, thereby enhancing their delivery to the intestinal tract [67]. In parallel, polysaccharides extracted from *Gracilaria fisheri* function as prebiotics, supplying fermentable substrates that foster a favorable gut environment for beneficial bacteria and contributing to immune modulation [68]. This co‐encapsulation approach not only preserves probiotic viability during gastrointestinal transit but also promotes microbial colonization and metabolic activity, thereby amplifying the probiotic’s beneficial effects on host health and performance. The synergistic interaction between alginate’s physical protection and the biochemical enhancement conferred by prebiotic polysaccharides creates an optimized delivery system that maximizes probiotic efficacy [25, 69].

Microbial analysis revealed marked alterations in the intestinal microbiota of treated fish. Populations of *Vibrio* spp., including both sucrose‐fermenting and nonfermenting strains, were significantly reduced in probiotic‐fed groups (*p* < 0.05), consistent with earlier studies demonstrating the antagonistic effects of probiotics against *Vibrio* pathogens in aquaculture [10]. The complete absence of *Aeromonas* spp. and *Pseudomonas* spp. in all experimental groups suggests a potential selective exclusion effect, likely mediated through competitive colonization or the production of antimicrobial metabolites [58]. Furthermore, the exclusive detection of *B. aryabhattai* in the intestines of probiotic‐treated fish confirms successful colonization and underscores its potential to promote host health via modulation of the intestinal microbial community.

Beyond growth and immune benefits, *B. aryabhattai* CKNJH11 supplementation also improved water quality. The D4 group exhibited significantly lower ammonia and nitrite concentrations, consistent with previous reports that *Bacillus* spp. can enhance water quality by metabolizing nitrogenous waste [70, 71]. Such environmental improvements reinforce the suitability of *B. aryabhattai* CKNJH11 as a sustainable probiotic, simultaneously supporting fish health and maintaining ecological balance in aquaculture systems.

Collectively, these findings indicate that dietary inclusion of *B. aryabhattai* CKNJH11, particularly when co‐encapsulated with prebiotic polysaccharides, can substantially improve growth performance, immune function, intestinal health, and environmental quality in *L. calcarifer* culture. This integrated strategy offers a promising, health‐conscious approach to sustainable aquaculture.

## 5. Conclusion

This study demonstrates that *B. aryabhattai* CKNJH11 is a safe and effective probiotic for Asian seabass, with its benefits maximized when administered as co‐encapsulated spores. The strain exhibited essential probiotic characteristics, including a nonhemolytic phenotype, high sporulation efficiency, strong tolerance to acidic and bile salt conditions, and biofilm inhibition against major aquaculture pathogens. Dietary supplementation enhanced growth performance, feed conversion efficiency, and hematological profiles in *L. calcarifer*, while reducing gut‐associated pathogenic *Vibrio* populations and lowering ammonia and nitrite concentrations, thereby improving water quality. Collectively, these results position co‐encapsulated *B. aryabhattai* CKNJH11 as a practical and environmentally sustainable solution for aquaculture health management, with the potential to reduce reliance on antibiotics. Future studies should investigate its long‐term efficacy, pathogen‐specific protective effects, and the molecular mechanisms underlying its immunomodulatory activity.

## Ethics Statement

All experiments in this study were performed in accordance with the relevant guidelines and regulations. The experimental protocols were approved by the Institutional Animal Care and Use Committee, Prince of Songkla University (Ref. AQ034/2025). All the procedure of the study is followed by the ARRIVE guidelines.

## Disclosure

A preprint of this work has been previously published (Appamano et al. [32]) and is available on Research Square (10.21203/rs.3.rs-6715276/v1).

## Conflicts of Interest

The authors declare no conflicts of interest.

## Author Contributions

**Waraporn Appamano:** methodology, investigation, writing – original draft. **Orathai Dangsawat:** methodology, investigation. **Sarayut Onsanit:** methodology, investigation **Rapeewan Sowanpreecha:** methodology, investigation. **Phatthanaphong Therdtatha:** writing – review & editing. **Tran Hoang Trieu Quan:** writing – review & editing. **Thi Hang Ho:** writing – review & editing. **Luu Tang Phuc Khang:** writing – review & editing. **Papungkorn Sangsawad:** writing – review & editing, formal analysis, supervision. **Nguyen Dinh-Hung:** formal analysis, writing – original draft, writing – review & editing. **Phan Do Trong Nghia:**, formal analysis, data curation, writing – review & editing. **Won-Kyo Jung:** writing – review & editing, formal analysis, supervision. **Nguyen Vu Linh:** investigation, methodology, validation, software, data curation, formal analysis, writing – review & editing. **Patima Permpoonpattana:** conceptualization, validation, resources, supervision, project administration, funding acquisition, and writing – review & editing.

## Funding

This project was financially supported by Prince of Songkla University, Surat Thani Campus, Thailand.

## Data Availability

The datasets used and/or analyzed during the current study are available from the corresponding author upon reasonable request.

## References

[1] FAO , The State of World Fisheries and Aquaculture, 2018, Food and Agriculture Organization of the United Nations.

[2] Pradeepkiran J. A. , Aquaculture Role in Global Food Security With Nutritional Value: A Review, Translational Animal Science. (2019) 3, no. 2, 903–910, 10.1093/tas/txz012, 2-s2.0-85072519769, 32704855. 32704855 PMC7200472

[3] Kemp J. O. , Taylor J. J. , Kelly L. A. , Larocque R. , Heriazon A. , Tiessen K. H. , and Cooke S. J. , Antibiotic Resistance Genes in the Aquaculture Sector: Global Reports and Research Gaps, Environmental Reviews. (2021) 29, no. 2, 300–314, 10.1139/er-2020-0087.

[4] Thornber K. , Bashar A. , Ahmed M. S. , Bell A. , Trew J. , Hasan M. , Hasan N. A. , Alam M. M. , Chaput D. L. , Haque M. M. , and Tyler C. R. , Antimicrobial Resistance in Aquaculture Environments: Unravelling the Complexity and Connectivity of the Underlying Societal Drivers, Environmental Science & Technology. (2022) 56, no. 21, 14891–14903, 10.1021/acs.est.2c00799. 36102785 PMC9631993

[5] Mohammed E. A. H. , Kovács B. , Kuunya R. , Mustafa E. O. A. , Abbo A. S. H. , and Pál K. , Antibiotic Resistance in Aquaculture: Challenges, Trends Analysis, and Alternative Approaches, Antibiotics. (2025) 14, no. 6, 10.3390/antibiotics14060598. PMC12189707 40558188

[6] Ghanei-Motlagh R. , Gharibi D. , Mohammadian T. , Khosravi M. , Mahmoudi E. , Zarea M. , Menanteau-Ledouble S. , and El-Matbouli M. , Feed Supplementation With Quorum Quenching Probiotics With Anti-Virulence Potential Improved Innate Immune Responses, Antioxidant Capacity and Disease Resistance in Asian Seabass (*Lates calcarifer*), Aquaculture. (2021) 535, 736345, 10.1016/j.aquaculture.2021.736345.

[7] Morshedi V. , Nafisi Bahabadi M. , Sotoudeh E. , Azodi M. , and Hafezieh M. , Nutritional Evaluation of *Gracilaria pulvinata* as Partial Substitute With Fish Meal in Practical Diets of Barramundi (*Lates calcarifer*), Journal of Applied Phycology. (2018) 30, no. 1, 619–628, 10.1007/s10811-017-1199-y, 2-s2.0-85021178339.

[8] Lan N. G. T. , Dong H. T. , Shinn A. P. , Vinh N. T. , Senapin S. , Salin K. R. , and Rodkhum C. , Review of Current Perspectives and Future Outlook on Bacterial Disease Prevention Through Vaccination in Asian Seabass (*Lates calcarifer*), Journal of Fish Diseases. (2024) 47, no. 8, e13964, 10.1111/jfd.13964. 38798108

[9] Yue G. and Guo C. , Strategies for Managing Major Diseases in Asian Seabass Aquaculture, Animal Diseases. (2025) 5, no. 1, 10.1186/s44149-025-00159-w.

[10] Kuebutornye F. K. , Abarike E. D. , Lu Y. , Hlordzi V. , Sakyi M. E. , Afriyie G. , Wang Z. , Li Y. , and Xie C. X. , Mechanisms and the Role of Probiotic *Bacillus* in Mitigating Fish Pathogens in Aquaculture, Fish Physiology and Biochemistry. (2020) 46, no. 3, 819–841, 10.1007/s10695-019-00754-y, 31953625. 31953625

[11] Soltani M. , Ghosh K. , Hoseinifar S. H. , Kumar V. , Lymbery A. J. , Roy S. , and Ringø E. , Genus *bacillus*, Promising Probiotics in Aquaculture: Aquatic Animal Origin, Bio-Active Components, Bioremediation and Efficacy in Fish and Shellfish, Reviews in Fisheries Science & Aquaculture. (2019) 27, no. 3, 331–379, 10.1080/23308249.2019.1597010, 2-s2.0-85065784003.

[12] Rahman M. M. , Paul S. I. , Rahman A. , Haque M. S. , Ador M. A. A. , Foysal M. J. , Islam M. T. , Rahman M. M. , Weththasinghe P. , and Rodriguez-Estrada U. , Suppression of Streptococcosis and Modulation of the Gut Bacteriome in Nile tilapia (*Oreochromis niloticus*) by the Marine Sediment Bacteria *Bacillus haynesii* and *Advenella mimigardefordensis* , Microbiology Spectrum. (2022) 10, no. 6, e02542, 10.1128/spectrum.02542-22. 36453920 PMC9769507

[13] Gao Y. , Tan R. , Wang Z. , Qiang L. , and Yao H. , The Effects of *Bacillus subtilis* on the Immunity, Mucosal Tissue Morphology, Immune-Related Gene Transcriptions, and Intestinal Microbiota in Flounder (*Paralichthys olivaceus*) With Two Feeding Methods: Continuous Versus Discontinuous Feeding, Veterinary Immunology and Immunopathology. (2024) 271, 110742, 10.1016/j.vetimm.2024.110742. 38547603

[14] Bondad-Reantaso M. G. , MacKinnon B. , Karunasagar I. , Fridman S. , Alday-Sanz V. , Brun E. , Le Groumellec M. , Li A. , Surachetpong W. , and Karunasagar I. , Review of Alternatives to Antibiotic Use in Aquaculture, Reviews in Aquaculture. (2023) 15, no. 4, 1421–1451, 10.1111/raq.12786.

[15] Anyairo C. S. , Unban K. , Shetty K. , and Khanongnuch C. , Bacteriocin Producing *Bacillus* and Their Potential Applications in Fish Farming, International Aquatic Research. (2024) 16, no. 1.

[16] Sachdeva A. , Tomar T. , Malik T. , Bains A. , and Karnwal A. , Exploring Probiotics as a Sustainable Alternative to Antimicrobial Growth Promoters: Mechanisms and Benefits in Animal Health, Frontiers in Sustainable Food Systems. (2025) 8, 1523678, 10.3389/fsufs.2024.1523678.

[17] Bhattacharyya C. , Bakshi U. , Mallick I. , Mukherji S. , Bera B. , and Ghosh A. , Genome-Guided Insights Into the Plant Growth Promotion Capabilities of the Physiologically Versatile *Bacillus aryabhattai* Strain AB211, Frontiers in Microbiology. (2017) 8, 10.3389/fmicb.2017.00411, 2-s2.0-85016544840. PMC5359284 28377746

[18] Park Y.-G. , Mun B.-G. , Kang S.-M. , Hussain A. , Shahzad R. , Seo C.-W. , Kim A.-Y. , Lee S.-U. , Oh K. Y. , Lee D. Y. , Lee I. J. , and Yun B. W. , *Bacillus aryabhattai* SRB02 Tolerates Oxidative and Nitrosative Stress and Promotes the Growth of Soybean by Modulating the Production of Phytohormones, PLoS One. (2017) 12, no. 3, e0173203, 10.1371/journal.pone.0173203, 2-s2.0-85015274135. 28282395 PMC5345817

[19] Tepaamorndech S. , Chantarasakha K. , Kingcha Y. , Chaiyapechara S. , Phromson M. , Sriariyanun M. , Kirschke C. P. , Huang L. , and Visessanguan W. , Effects of *Bacillus aryabhattai* TBRC8450 on Vibriosis Resistance and Immune Enhancement in Pacific White Shrimp, *Litopenaeus vannamei* , Fish & Shellfish Immunology. (2019) 86, 4–13, 10.1016/j.fsi.2018.11.010, 2-s2.0-85056474126, 30419397. 30419397

[20] Dangsawat O. , Rattanawut J. , Srisawat T. , Sowanpreecha R. , Tang Phuc Khang L. , Srinual O. , Hung N. D. , do-Hyung K. , Husna N. N. , Dwinanti S. H. , Linh N. V. , and Permpoonpattana P. , *Bacillus aryabhattai* CKNJh11 as a Promising Probiotic Improves Growth Performance and Egg Quality in Laying Hens, Scientific Reports. (2025) 15, no. 1, 10.1038/s41598-025-97553-8. PMC12009989 40254647

[21] Fachri M. , Amoah K. , Huang Y. , Cai J. , Alfatat A. , Ndandala C. B. , Shija V. M. , Jin X. , Bissih F. , and Chen H. , Probiotics and Paraprobiotics in Aquaculture: a Sustainable Strategy for Enhancing Fish Growth, Health and Disease Prevention-A Review, Frontiers in Marine Science. (2024) 11, 1499228, 10.3389/fmars.2024.1499228.

[22] Wang Y.-B. , Li J.-R. , and Lin J. , Probiotics in Aquaculture: Challenges and Outlook, Aquaculture. (2008) 281, no. 1-4, 1–4, 10.1016/j.aquaculture.2008.06.002, 2-s2.0-48349094063.

[23] Merrifield D. L. and Ringo E. , Aquaculture Nutrition: Gut Health, Probiotics and Prebiotics, 2014, John Wiley & Sons, 10.1002/9781118897263.

[24] Vivek K. , Mishra S. , Pradhan R. C. , Nagarajan M. , Kumar P. K. , Singh S. S. , Manvi D. , and Gowda N. N. , A Comprehensive Review on Microencapsulation of Probiotics: Technology, Carriers and Current Trends, Applied Food Research. (2023) 3, no. 1, 100248, 10.1016/j.afres.2022.100248.

[25] Agriopoulou S. , Tarapoulouzi M. , Varzakas T. , and Jafari S. M. , Application of Encapsulation Strategies for Probiotics: From Individual Loading to Co-Encapsulation, Microorganisms. (2023) 11, no. 12, 10.3390/microorganisms11122896. PMC10745938 38138040

[26] Ramadhani D. E. , Widanarni W. , and Sukenda S. , Microencapsulation of Probiotics and its Applications With Prebiotic in Pacific White Shrimp Larvae Through *Artemia* sp, Jurnal Akuakultur Indonesia. (2019) 18, no. 2, 130–140, 10.19027/jai.18.2.130-140.

[27] Nezamdoost-Sani N. , Khaledabad M. A. , Amiri S. , and Khaneghah A. M. , Alginate and Derivatives Hydrogels in Encapsulation of Probiotic Bacteria: An Updated Review, Food Bioscience. (2023) 52, 102433, 10.1016/j.fbio.2023.102433.

[28] de Araújo Etchepare M. , Barin J. S. , Cichoski A. J. , Jacob-Lopes E. , Wagner R. , Fries L. L. M. , and de Menezes C. , Microencapsulation of probiotics using sodium alginate, Ciência Rural. (2015) 45, no. 7, 1319–1326, 10.1590/0103-8478cr20140938, 2-s2.0-84932649968.

[29] Hayisama-Ae W. , Kantachote D. , Bhongsuwan D. , Nokkaew U. , and Chaiyasut C. , A Potential Synbiotic Beverage From Fermented Red Seaweed (*Gracilaria fisheri*) Using *Lactobacillus plantarum* DW12, International Food Research Journal. (2014) 21, no. 5.

[30] O’Sullivan L. , Murphy B. , McLoughlin P. , Duggan P. , Lawlor P. G. , Hughes H. , and Gardiner G. E. , Prebiotics From Marine Macroalgae for Human and Animal Health Applications, Marine Drugs. (2010) 8, no. 7, 2038–2064, 10.3390/md8072038, 2-s2.0-77955288665. 20714423 PMC2920542

[31] Praiboon J. , Chantorn S. , Krangkratok W. , Choosuwan P. , and La-Ongkham O. , Evaluating the Prebiotic Properties of Agar Oligosaccharides Obtained From the Red Alga *Gracilaria Fisheri* via Enzymatic Hydrolysis, Plants. (2023) 12, no. 23, 10.3390/plants12233958. PMC10708334 38068595

[32] Appamano W. , Dangsawat O. , Onsanit S. , Sowanpreecha R. , Therdtatha P. , Quan T. H. T. , Ho T. H. , Khang L. T. P. , Sangsawad P. , and Dinh-Hung N. , Co-Encapsulation of *Bacillus aryabhattai* CKNJH11 With Algae-Derived Polysaccharides Enhances Growth Performance and Immune Response in Asian Seabass (*Lates calcarifer*), Research Square. (2025) 10.21203/rs.3.rs-6715276/v1. PMC12752878 41476899

[33] Mwamburi S. M. , Islam S. I. , Dinh-Hung N. , Dangsawat O. , Sowanpreecha R. , Khang L. T. P. , Montha N. , Therdtatha P. , Dwinanti S. H. , Permpoonpattana P. , and Linh N. V. , Genomic Characterization of *Bacillus* sp. THPS1: A Hot Spring-Derived Species With Functional Features and Biotechnological Potential, Microorganisms. (2024) 12, no. 12, 10.3390/microorganisms12122476. PMC11727782 39770679

[34] Brosius J. , Dull T. J. , Sleeter D. D. , and Noller H. F. , Gene Organization and Primary Structure of a Ribosomal RNA Operon From *Escherichia coli* , Journal of Molecular Biology. (1981) 148, no. 2, 107–127, 10.1016/0022-2836(81)90508-8, 2-s2.0-0019513663. 7028991

[35] Katoh K. , Misawa K. , Kuma K. , and Miyata T. , MAFFT: A Novel Method for Rapid Multiple Sequence Alignment Based on Fast Fourier Transform, Nucleic Acids Research. (2002) 30, no. 14, 3059–3066, 10.1093/nar/gkf436. 12136088 PMC135756

[36] Minh B. Q. , Schmidt H. A. , Chernomor O. , Schrempf D. , Woodhams M. D. , Von Haeseler A. , and Lanfear R. , IQ-TREE 2: New Models and Efficient Methods for Phylogenetic Inference in the Genomic Era, Molecular Biology and Evolution. (2020) 37, no. 5, 1530–1534, 10.1093/molbev/msaa015. 32011700 PMC7182206

[37] Li Z. , Siepmann F. B. , Tovar L. E. R. , Chen X. , and Gänzle M. G. , Effect of Copy Number of the *spoVA* ^2mob^ Operon, Sourdough and reuteriCyclin on Ropy Bread Spoilage Caused by *Bacillus* spp., Food Microbiology. (2020) 91, 103507, 10.1016/j.fm.2020.103507. 32539950

[38] Hamza F. , Kumar A. R. , and Zinjarde S. , Antibiofilm Potential of a Tropical Marine *Bacillus licheniformis* isolate: Role in Disruption of Aquaculture Associated Biofilms, Aquaculture Research. (2016) 47, no. 8, 2661–2669, 10.1111/are.12716, 2-s2.0-84922902177.

[39] Anand C. , Gordon R. , Shaw H. , Fonseca K. , and Olsen M. , Pig and Goat Blood as Substitutes for Sheep Blood in Blood-Supplemented Agar Media, Journal of Clinical Microbiology. (2000) 38, no. 2, 591–594, 10.1128/JCM.38.2.591-594.2000. 10655351 PMC86154

[40] Dinh-Hung N. , Dong H. T. , Senapin S. , Pimsannil K. , Thompson K. D. , Shinn A. P. , Soontara C. , Sirimanapong W. , Chatchaiphan S. , and Rodkhum C. , Insight Into Characteristics and Pathogenicity of Five Rapidly Growing Non-Tuberculous *Mycobacterium* Species Isolated From the Siamese Fighting Fish, *Betta splendens* , Aquaculture. (2023) 575, 739822, 10.1016/j.aquaculture.2023.739822.

[41] Goncalves G. , Santos R. A. , Coutinho F. , Pedrosa N. , Curado M. , Machado M. , Costas B. , Bonneville L. , Serrano M. , and Carvalho A. P. , Oral Vaccination of Fish Against Vibriosis Using Spore-Display Technology, Frontiers in Immunology. (2022) 13, 10.3389/fimmu.2022.1012301. PMC9608137 36311700

[42] Linh N. V. , Khongcharoen N. , Nguyen D.-H. , Dien L. T. , Rungrueng N. , Jhunkeaw C. , Sangpo P. , Senapin S. , Uttarotai T. , Panphut W. , St-Hilaire S. , van Doan H. , and Dong H. T. , Effects of Hyperoxia During Oxygen Nanobubble Treatment on Innate Immunity, Growth Performance, Gill Histology, and Gut Microbiome in Nile tilapia, Oreochromis niloticus, Fish & Shellfish Immunology. (2023) 143, 109191, 10.1016/j.fsi.2023.109191. 37890736

[43] Sintuprom C. , Nuchchanart W. , Dokkaew S. , Aranyakanont C. , Ploypan R. , Shinn A. P. , Wongwaradechkul R. , Dinh-Hung N. , Dong H. T. , and Chatchaiphan S. , Effects of Confinement Rearing and Sodium Chloride Treatment on Stress Hormones and Gene Expression in Siamese Fighting Fish (*Betta splendens*), Journal of Applied Animal Welfare Science. (2025) 1–17, 10.1080/10888705.2025.2481884, 40137956. 40137956

[44] Sintuprom C. , Nuchchanart W. , Dokkaew S. , Aranyakanont C. , Ploypan R. , Shinn A. P. , Wongwaradechkul R. , Dinh-Hung N. , Dong H. T. , and Chatchaiphan S. , Effects of Clove Oil Concentrations on Blood Chemistry and Stress-Related Gene Expression in Siamese Fighting Fish (*Betta splendens*) During Transportation, Frontiers in Veterinary Science. (2024) 11, 1392413, 10.3389/fvets.2024.1392413. 38840639 PMC11151877

[45] Ramlucken U. , Ramchuran S. O. , Moonsamy G. , van Rensburg C. J. , Thantsha M. S. , and Lalloo R. , Production and Stability of a Multi-Strain Bacillus Based Probiotic Product for Commercial Use in Poultry, Biotechnology Reports. (2021) 29, e00575, 10.1016/j.btre.2020.e00575. 33659192 PMC7890156

[46] Yufera M. , Moyano F. J. , Astola A. , Pousao-Ferreira P. , and Martinez-Rodriguez G. , Acidic Digestion in a Teleost: Postprandial and Circadian Pattern of Gastric pH, Pepsin Activity, and Pepsinogen and Proton Pump mRNAs Expression, PLoS One. (2012) 7, no. 3, e33687, 10.1371/journal.pone.0033687, 2-s2.0-84858650303. 22448266 PMC3309002

[47] Petit C. , Caudal F. , Taupin L. , Dufour A. , Le Ker C. , Giudicelli F. , Rodrigues S. , and Bazire A. , Antibiofilm Activity of the Marine Probiotic *Bacillus subtilis* C3 Against the Aquaculture-Relevant Pathogen *Vibrio harveyi* , Probiotics and Antimicrobial Proteins. (2025) 17, no. 3, 1551–1562, 10.1007/s12602-024-10229-z. 38329698

[48] Hau T. H. , Van T. T. B. , Quynh C. N. T. , Oanh T. T. H. , Huy L. A. G. , Luu N. H. , and Thi N. P. A. , Inhibition of Biofilm-Forming Bacteria and Probiotic Potential of *Bacillus* spp. Isolated From Aquaculture Ponds, Malaysian Journal of Microbiology. (2024) 20, no. 4, 10.21161/mjm.230403.

[49] Prazdnova E. , Zaikina A. , Neurov A. , Mazanko M. , Ranjan A. , and Rudoy D. , Bacillibactin, a Potential *Bacillus*-Based Antibacterial Non-Ribosomal Peptide: In Silico Studies for Targeting Common Fish Pathogens, International Journal of Molecular Sciences. (2025) 26, no. 12, 10.3390/ijms26125811, 40565273. PMC12192977 40565273

[50] Atef S. , Ahmed O. M. , Said M. M. , and Abo-Al-Ela H. G. , Dietary *Bacillus* Species Modulate Lipid Metabolism-Related Parameters, Growth, Water Quality, and Bacterial Load in Nile Tilapia (*Oreochromis niloticus*), Animal Feed Science and Technology. (2024) 310, 115943, 10.1016/j.anifeedsci.2024.115943.

[51] Dighiesh H. S. , Alharbi N. A. , Awlya O. F. , Alhassani W. E. , Hassoubah S. A. , Albaqami N. M. , Aljahdali N. , El-Aziz A. , Yasmin M. , and Eissa E.-S. H. , Dietary Multi-Strains *Bacillus* spp. Enhanced Growth Performance, Blood Metabolites, Digestive Tissues Histology, Gene Expression of *Oreochromis niloticus*, and Resistance to *Aspergillus flavus* Infection, Aquaculture International. (2024) 32, no. 6, 7065–7086, 10.1007/s10499-024-01502-7.

[52] Luo K. , Guo Z. , Liu Y. , Li C. , Ma Z. , and Tian X. , Responses of Growth Performance, Immunity, Disease Resistance of Shrimp and Microbiota in *Penaeus vannamei* Culture System to *Bacillus subtilis* BSXE-1601 Administration: Dietary Supplementation *Versus* Water Addition, Microbiological Research. (2024) 283, 127693, 10.1016/j.micres.2024.127693. 38490029

[53] Pan M. V. , Ferriols V. M. E. N. , and Traifalgar R. F. M. , Synergistic Influence of Hydrolyzed Squid Processing By-Products and *Bacillus* Probiotics as Dietary Supplements on Growth Performance, Immunological Responses, and Gut Health of Juvenile Black Tiger Shrimp Fed Fishmeal-Free Diets, Aquaculture International. (2024) 32, no. 4, 4551–4580, 10.1007/s10499-024-01390-x.

[54] Keereelang J. , Mangumphan K. , Chitmanat C. , Tongsiri S. , Linh N. V. , and Van Doan H. , Dietary Effect of *Lactobacillus plantarum* (TISTR 912) on Digestive Enzyme Activity, Growth Performance, Immune Response, and Disease Resistance of Black Sharkminnow (*Labeo chrysophekadion*) Against *Aeromonas hydrophila* Infection, Aquaculture Reports. (2022) 27, 101409, 10.1016/j.aqrep.2022.101409.

[55] Aini N. , Putri D. S. Y. R. , Achhlam D. H. , Fatimah F. , Andriyono S. , Hariani D. , Do H. D. K. , and Wahyuningsih S. P. A. , Supplementation of *Bacillus subtilis* and *Lactobacillus casei* to increase growth performance and immune system of catfish (*Clarias gariepinus*) due to *Aeromonas hydrophila* infection, Veterinary World. (2024) 17, no. 3, 602–611, 10.14202/vetworld.2024.602-611, 38680146. 38680146 PMC11045519

[56] Liaqat R. , Fatima S. , Komal W. , Minahal Q. , Kanwal Z. , Suleman M. , and Carter C. G. , Effects of *Bacillus subtilis* as a Single Strain Probiotic on Growth, Disease Resistance and Immune Response of Striped Catfish (*Pangasius hypophthalmus*), PLoS One. (2024) 19, no. 1, e0294949, 10.1371/journal.pone.0294949. 38289940 PMC10842300

[57] Zhang Q. , Ma H. , Mai K. , Zhang W. , Liufu Z. , and Xu W. , Interaction of Dietary *Bacillus subtilis* and Fructooligosaccharide on the Growth Performance, Non-Specific Immunity of Sea Cucumber, *Apostichopus japonicus* , Fish & Shellfish Immunology. (2010) 29, no. 2, 204–211, 10.1016/j.fsi.2010.03.009, 2-s2.0-77954957795. 20371291

[58] Nayak S. K. , Multifaceted Applications of Probiotic *Bacillus* species in Aquaculture With Special Reference to *Bacillus subtilis* , Reviews in Aquaculture. (2021) 13, no. 2, 862–906, 10.1111/raq.12503.

[59] Ma S. , Yu D. , Liu Q. , Zhao M. , Xu C. , and Yu J. , Relationship Between Immune Performance and the Dominant Intestinal Microflora of Turbot Fed With Different *Bacillus* Species, Aquaculture. (2022) 549, 737625, 10.1016/j.aquaculture.2021.737625.

[60] Zhang J. , Huang M. , Feng J. , Chen Y. , Li M. , and Chang X. , Effects of Dietary *Bacillus licheniformis* on Growth Performance, Intestinal Morphology, Intestinal Microbiome, and Disease Resistance in Common Carp (*Cyprinus carpio* L.), Aquaculture International. (2021) 29, no. 3, 1343–1358, 10.1007/s10499-021-00701-w.

[61] Shija V. M. , Amoah K. , and Cai J. , Effect of *Bacillus* Probiotics on the Immunological Responses of Nile Tilapia (*Oreochromis niloticus*): A Review, Fishes. (2023) 8, no. 7, 10.3390/fishes8070366.

[62] Nakharuthai C. , Boonanuntanasarn S. , Kaewda J. , and Manassila P. , Isolation of Potential Probiotic *Bacillus* spp. From the Intestine of Nile Tilapia to Construct Recombinant Probiotic Expressing CC Chemokine and its Effectiveness on Innate Immune Responses in Nile Tilapia, Animals. (2023) 13, no. 6, 10.3390/ani13060986. PMC10044694 36978530

[63] Newaj-Fyzul A. , Adesiyun A. A. , Mutani A. , Ramsubhag A. , Brunt J. , and Austin B. , *Bacillus subtilis* AB1 Controls Aeromonas Infection in Rainbow Trout (*Oncorhynchus mykiss*, Walbaum), Journal of Applied Microbiology. (2007) 103, no. 5, 1699–1706, 10.1111/j.1365-2672.2007.03402.x, 2-s2.0-35448985821. 17953580

[64] Irianto A. and Austin B. , Use of Probiotics to Control Furunculosis in Rainbow Trout, *Oncorhynchus mykiss* (Walbaum), Journal of Fish Diseases. (2002) 25, no. 6, 333–342, 10.1046/j.1365-2761.2002.00375.x, 2-s2.0-0036063699. 12962213

[65] Nayak S. , Swain P. , and Mukherjee S. , Effect of Dietary Supplementation of Probiotic and Vitamin C on the Immune Response of Indian Major Carp, *Labeo rohita* (Ham.), Fish & Shellfish Immunology. (2007) 23, no. 4, 892–896, 10.1016/j.fsi.2007.02.008, 2-s2.0-34547637287. 17434319

[66] Amenyogbe E. , Zhang J. D. , Huang J. S. , and Chen G. , The Efficiency of Indigenous Isolates *Bacillus* sp. RCS1 and *Bacillus* cereus RCS3 on Growth Performance, Blood Biochemical Indices and Resistance Against *Vibrio harveyi* in Cobia Fish (*Rachycentron canadum*) Juveniles, Aquaculture Reports. (2022) 25, 101241, 10.1016/j.aqrep.2022.101241.

[67] Benn J. S. , Chaki S. P. , Xu Y. , Ficht T. A. , Rice-Ficht A. C. , and Cook W. E. , Protective Antibody Response Following Oral Vaccination With Microencapsulated *Bacillus Anthracis* Sterne Strain 34F2 Spores, npj Vaccines. (2020) 5, no. 1, 10.1038/s41541-020-0208-3. PMC7351773 32685200

[68] Liu M. , Sun C. , Zhou Q. , Xu P. , Wang A. , Zheng X. , and Liu B. , Supplementation of Yupingfeng Polysaccharides in Low Fishmeal Diets Enhances Intestinal Health Through Influencing the Intestinal Barrier, Immunity, and Microflora in *Macrobrachium rosenbergii* , Frontiers in Immunology. (2024) 15, 1480897, 10.3389/fimmu.2024.1480897. 39660141 PMC11628508

[69] Madybekova G. , Turkeyeva E. , Mutaliyeva B. , Osmanova D. , Aidarova S. , Miller R. , Sharipova A. , and Issayeva A. , Study of Probiotic Bacteria Encapsulation for Potential Application in Enrichment of Fermented Beverage, Colloids and Interfaces. (2024) 8, no. 5, 10.3390/colloids8050051.

[70] Lalloo R. , Ramchuran S. , Ramduth D. , Görgens J. , and Gardiner N. , Isolation and Selection of *Bacillus* spp. as Potential Biological Agents for Enhancement of Water Quality in Culture of Ornamental Fish, Journal of Applied Microbiology. (2007) 103, no. 5, 1471–1479, 10.1111/j.1365-2672.2007.03360.x, 2-s2.0-35448994450. 17953558

[71] Thurlow C. M. , Williams M. A. , Carrias A. , Ran C. , Newman M. , Tweedie J. , Allison E. , Jescovitch L. N. , Wilson A. E. , Terhune J. S. , and Liles M. R. , *Bacillus velezensis* AP193 Exerts Probiotic Effects in Channel Catfish (*Ictalurus punctatus*) and Reduces Aquaculture Pond Eutrophication, Aquaculture. (2019) 503, 347–356, 10.1016/j.aquaculture.2018.11.051, 2-s2.0-85060094554.

